# Angiogenesis in Breast Cancer Progression, Diagnosis, and Treatment

**DOI:** 10.7150/jca.44313

**Published:** 2020-05-18

**Authors:** Chikezie O. Madu, Stephanie Wang, Chinua O. Madu, Yi Lu

**Affiliations:** 1 Departments of Biological Sciences, University of Memphis, Memphis, TN 38152. USA.; 2 Departments of Biology and Advanced Placement Biology, White Station High School, Memphis, TN 38117. USA.; 3 Departments of Biology and Advanced Placement Biology, White Station High School, Memphis, TN 38117. USA.; 4 Department of Pathology and Laboratory Medicine, University of Tennessee Health Science Center, Memphis, TN 38163. USA.

**Keywords:** angiogenesis, VEGF, Breast cancer, metastasis, angiogenesis, hypoxia, vascular diseases, anti-angiogenic therapies

## Abstract

Angiogenesis is a significant event in a wide range of healthy and diseased conditions. This process frequently involves vasodilation and an increase in vascular permeability. Numerous players referred to as angiogenic factors, work in tandem to facilitate the outgrowth of endothelial cells (EC) and the consequent vascularity. Conversely, angiogenic factors could also feature in pathological conditions.

Angiogenesis is a critical factor in the development of tumors and metastases in numerous cancers. An increased level of angiogenesis is associated with decreased survival in breast cancer patients. Therefore, a good understanding of the angiogenic mechanism holds a promise of providing effective treatments for breast cancer progression, thereby enhancing patients' survival. Disrupting the initiation and progression of this process by targeting angiogenic factors such as vascular endothelial growth factor (Vegf)-one of the most potent member of the VEGF family- or by targeting transcription factors, such as Hypoxia-Inducible Factors (HIFs) that act as angiogenic regulators, have been considered potential treatment options for several types of cancers.

The objective of this review is to highlight the mechanism of angiogenesis in diseases, specifically its role in the progression of malignancy in breast cancer, as well as to highlight the undergoing research in the development of angiogenesis-targeting therapies.

## Introduction

According to the American Cancer Society in 2018, breast cancer (BCa) is the most common invasive malignancy and the second leading cause of tumor-related death among women globally. It is estimated that about 270,000 new cases of invasive breast cancer are projected to be diagnosed in women in the U.S. in 2018. 1 In the U.S., breast cancer death rates are higher than those of any other cancer, except for lung and skin cancer. Furthermore, it is the most commonly diagnosed cancer among American women, 2 with 40,290 women estimated to die from breast cancer annually. In contrast, only about 2,500 new cases of invasive breast cancer are expected to be diagnosed in men; 440 of that number will die from the disease. In women under 45, breast cancer is more prevalent in African-American women than in Caucasian women. African-American women overall, are more likely to die of breast cancer than women of other ethnicities, although the five-year relative survival rate for women with invasive breast cancer surged from 75 percent to 90 percent in a twenty-year period. 3 The mortality rate has dropped nearly 40% in the past 25 years due to a combination of improved early diagnosis and advanced medical treatment. 2

Angiogenesis, the rapid increase in the formation of blood vessels, is required for supply of sufficient oxygen and nutrition for breast tumor growth. Breast cancer cells, like all body tissues, need constant nourishment and oxygen supply through the vascular network of capillaries in the system. 4 These capillaries usually do not proliferate because the cells that line the interior surface of blood vessels, endothelial cells (ECs), do not multiply. Low levels of O2 (hypoxia) triggers numerous transcriptional responses, mediated by transcription factors, referred to as hypoxia-inducible factors (HIFs). HIFs are highly conserved transcription factors that regulate the expression of multiple genes responsible for stimulating specific physiological responses, such as metabolism, angiogenesis, and cell division. Local angiogenesis is one of the tumor's microenvironment long-term primary adaptation to low O2 levels. 5 It involves the convergence of EC precursors that give rise to capillary plexus, subsequently developing into blood vessels. Angiogenesis is a crucial player in normal processes, such as embryonic development, growth, and wound healing. 6

Angiogenesis under physiological circumstances involves the generation of novel ECs and the subsequent structural morphing of them into tubes. 7 Angiogenesis is critical in the development, progression, and metastasis of solid tumor cells. 8 During its onset, the tumor does not stimulate angiogenesis, and with low nutrient and oxygen supply, will remain limited in its growth to about 1-2 mm in diameter. 9,10 In this early phase, the tumor may reside in a dormant state, where the rate of cell death counterbalances cell proliferation, partly due to the hypoxia and, hence, insufficiently available nutrients in the microenvironment. This condition is due to the demand created by metabolites produced by the tumor cells. 11

Consequently, the tumor activates an angiogenic switch and evolves irreversibly to an active angiogenic state. 12 This newly attained status by the tumor confers upon it the ability to recruit new capillaries, thereby resuming oxygen and nutrients supplies to both the angiogenic cells and the surrounding non-angiogenic cells, leading to rapidly increasing tumor growth. 13 Although surgically removing tumors is the current primary treatment of breast cancer, adjuvant treatment such as anti-angiogenic therapy has been employed after surgery, in advanced disease stages, when the option of surgery is no longer available. 14,15

The initiation and progression of tumor angiogenesis are mainly due to angiogenic growth factors, such as vascular endothelial growth factor (VEGF) and fibroblast growth factors (FGF). 16-19 Several studies have shown that levels of angiogenic factors, and the subsequent number of vascular networks formed, is a predictive factor for breast cancer survival. 20-22 In other words, elevated levels are symptomatic of the aggressive nature of the respective tumor cells and correlate to a relatively poor prognosis. 23 -25 Coupled with activating angiogenesis, these factors also dictate the rate and extent to which the blood vessels permeate. To this end, compounds that target the angiogenesis pathway have increasingly attracted attention in research in breast cancer therapy. 26

The most extensively studied compound is the drug, Bevacizumab, a humanized anti-VEGF monoclonal antibody. The FDA approved bevacizumab in 2008 for treatment of metastatic HER2-negative breast cancer after promising results in targeting VEGF were observed in preclinical trials. 27 Following that, several anti-angiogenic drugs targeting VEGF or blocking the activity of its receptor, have been approved, and are commonly used in the treatment of different cancers. 28-29 In 2011, however, the FDA rescinded its approval due to contradictory results from previous studies and reports of resultant elevated toxicity. 31,32

While the discovery of these anti-angiogenic drugs and small molecules were hailed as a breakthrough and potential victory in one aspect of the fight against cancer, this celebration was quelled by the modest activities of these agents, such as their inability to arrest recurrent tumors in a latent state, and the moderate improvement they provide in overall patient survival.

## Mechanisms of Angiogenesis

The growth and metastasis of tumors largely depend on angiogenesis. 13,17 When blood supply is deficient, tumors are incapable of growing, necrosis sets. After a while, a subsequent metastatic spread to the systemic circulation is prevented. 8, 33 Research of the mechanism and the various factors surrounding angiogenesis have helped scientists understand its impact on breast cancer and mount a countermeasure against tumor progression. Due to the dual nature of this process, however, it is vital to carefully analyze and distinguish between the mechanism that leads to normal angiogenesis, such as wound repair, normal growth, and embryo nourishment, and that of tumor-related angiogenesis.

Certain substances, referred to as angiogenic activators due to their capability of stimulating proliferation of different cells in vitro, are responsible for the initiation of angiogenesis, 17, 25 which involves signaling between tumor cells and numerous other cell types within the tumor microenvironment.

The induction of this process has been shown to depend on the production of pro-angiogenic growth factors by the tumor cells, which affect the existing vessels. 13 During these tightly regulated processes, a complex signal balance, between pro- and anti-angiogenic factors, is aggressively sustained in the microenvironment, to develop and stabilize the newly formed blood vessels. 34 Numerous studies, therefore, have confirmed that these angiogenic activators play an essential role in the development of tumors. 35

**Figure 1:**
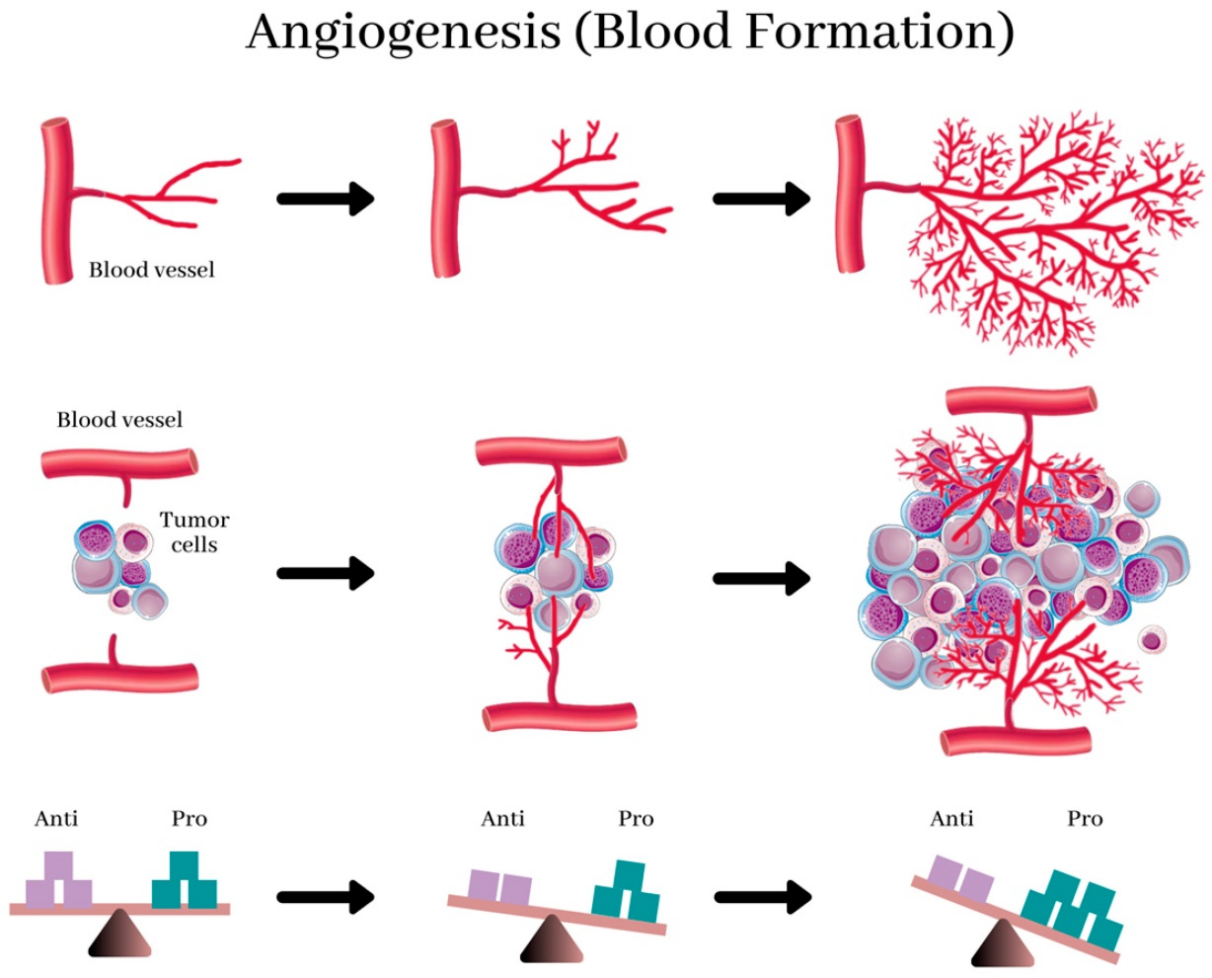
Angiogenesis, the physiological process by which development of new blood vessels from preexisting vessels. New blood vessels form out of pre-existing capillaries. The new blood vessels, near and in the tumor, provide it with essential nutrients for growth. Angiogenesis in healthy tissues is regulated by the balance between anti- and pro-angiogenic factors (bottom), and this balance is destroyed by the prevalence of angiogenic factors in tumors, resulting in abnormal structure and function of blood vessels and leading to hypoxia. This reverts the balance and normalizes the vasculature. 37

Studies that were done earlier revealed that specific tumor cells produce both pro- and anti-angiogenic proteins that stimulate and inhibits angiogenesis, respectively. 11 (Figure 1) 36 Scientists believe that tumors activate the angiogenic switch by altering the balance between angiogenesis inducers and inhibitors exerting opposing action. 13 This switch can be accomplished by changing the transcription of the genes as observed in several tumors where an increase in the levels of VEGF and/or FGFs is recorded when compared to healthy tissue. Conversely, in other tumors, the levels of endogenous inhibitors are reduced. 37 However, the complex mechanism that directs these shifts in the balances between the regulators is still a subject of fascinating study.

**Figure 2:**
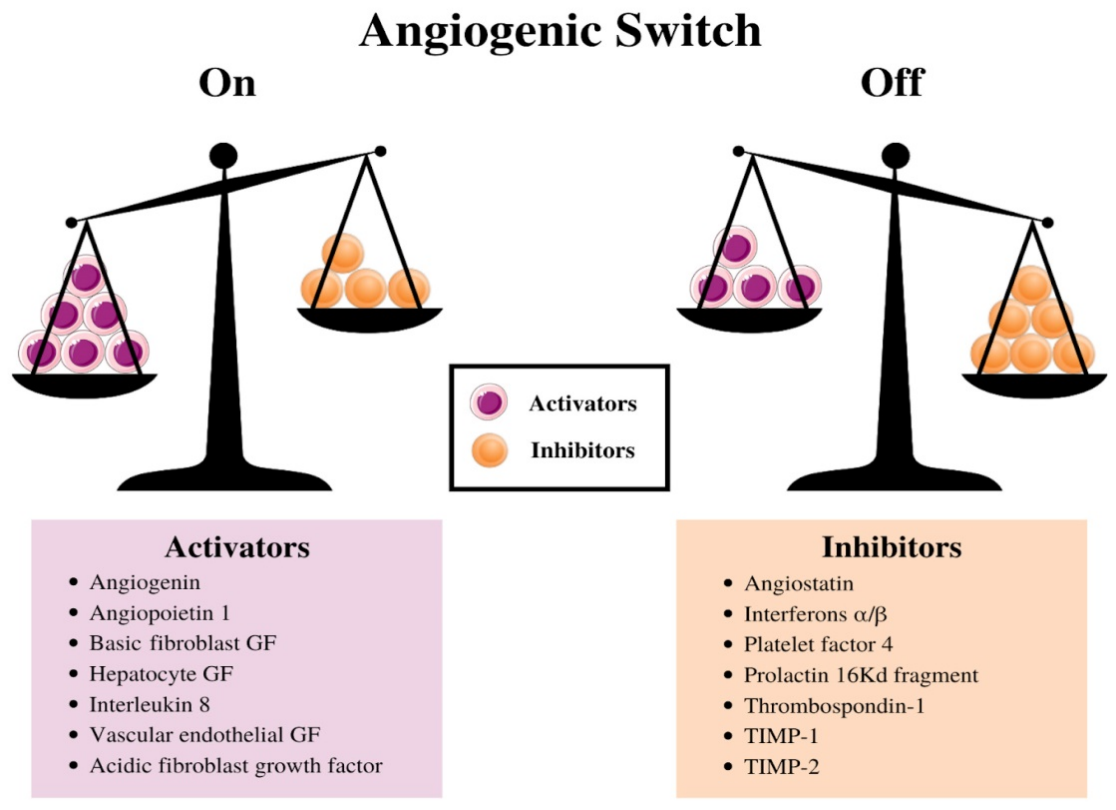
Schematic diagram illustrating the balance hypothesis of the angiogenic switch. It is speculated that an angiogenesis switch mechanism tightly regulates normal angiogenesis (formation of new capillaries). This balance can be disrupted to favor increased blood vessel formation through inducers and inhibitors of angiogenesis, which activates the switch. Reducing the inhibitor concentration, e.g., thrombospondin-1, 16kD prolactin, Interferon αιβ, Platelet factor-4, Angiostatin, etc. or increasing the activator levels, e.g., aFGF, bFGF, VEGF, etc., can change the balance and activate the switch, which could lead to the growth of new blood vessels. 13

The balance between this switch determines whether the tumor will switch on angiogenesis. 13 (Figure 2). 38 Further experiments indicated a decrease in the production of the anti-angiogenic proteins turns on the tumor angiogenic switch, 39 and consequently, promotes the tumor growth and metastases. 40-42 Stimulating angiogenesis in a tumor and creating the subsequent endothelial tubes involves a multistep process that is regulated by hypoxia at every step. This process relies extensively on ECs expressing the heterodimeric transcription factor, HIF-1α 43. HIF-1α protein is stabilized and forms a heterodimer with HIF-1β under hypoxic conditions, 43,44 and this duo activates the transcription of several target genes to adapt to the hypoxic environment in human cancer cells. 45

Some studies have shown that HIF-1α, working in tandem with other members of the HIF family, regulates nearly every aspect of angiogenesis, thereby making the HIF pathway a master regulator of angiogenesis. HIF-1α and HIF-2α expression have also been associated with poor prognosis and metastatic disease in several cancers 46. As a result, it is considered an attractive therapeutic target for many diseases. 47,48

For the new blood vessels to sprout and grow, hypoxia and the HIF pathway activation in the tumor cells are critical, since they regulate the expression of a collection of pro-angiogenic genes These includes the potent cytokines, vascular endothelial growth factor (VEGF)—an endothelial mitogen and pro-angiogenic factor, 39,49,50 angiopoietin-1, angiopoietin-2, platelet-derived growth factor (PDGF), and basic fibroblast growth factor (bFGF). 51

Additional research has centered more on the FGF and VEGF families 52 than all other angiogenetic growth factors. 53 Vascular endothelial growth factor (VEGF-A) was discovered in 1983 and sequenced completely in 1989. It was the first cytokine characterized as a major contributor to tumor angiogenesis, 52,54,55 was originally purified from tumor cell ascites as vascular permeability factor (VPF), 53 and also reported to have some biological effects on EC mitogenesis; thus, VPF is generally referred to as VEGF. 54,56,57

**Figure 3:**
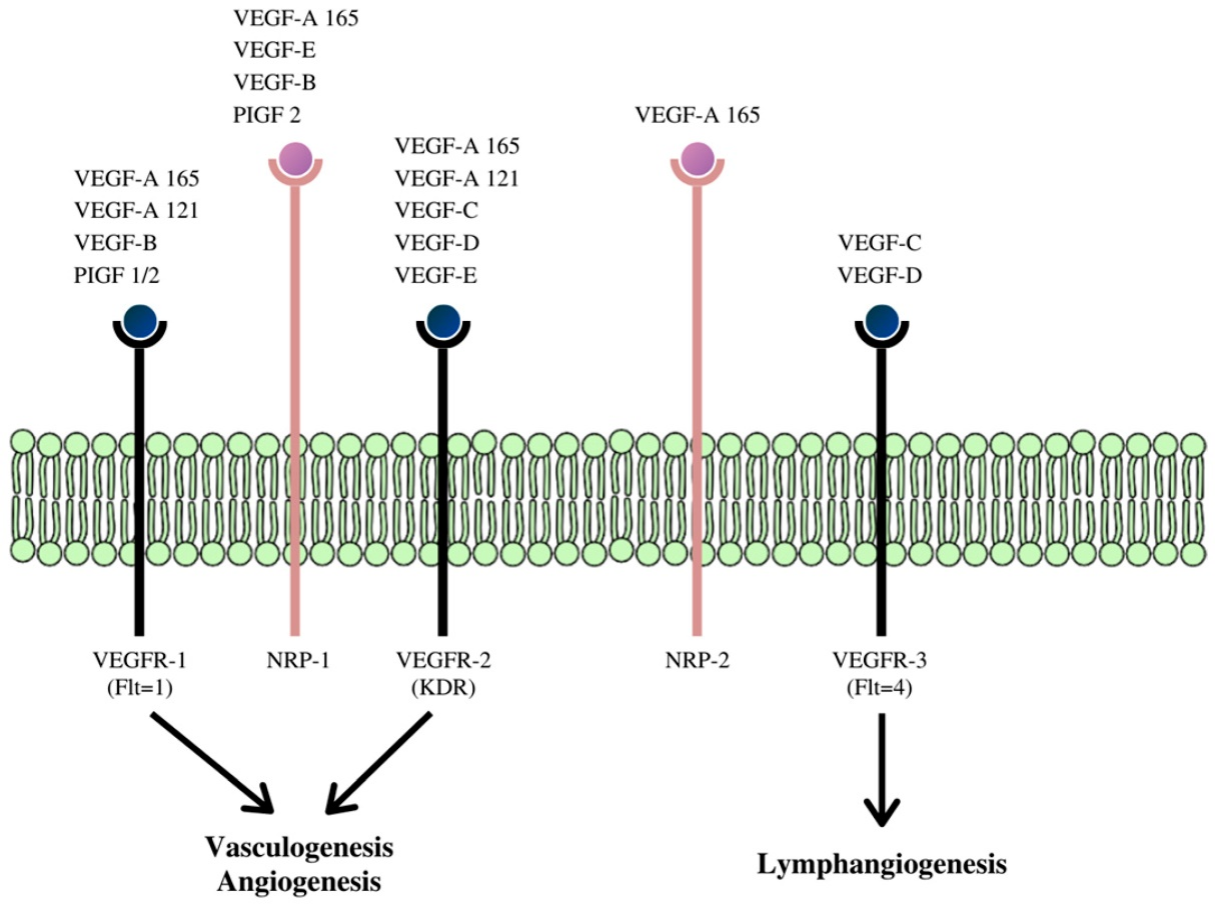
Schematic diagram of the receptor-binding specificity of vascular endothelial growth factor (VEGF) family members and their signaling pathways. VEGF family members bind to specific receptor tyrosine kinases: VEGFR-1, VEGFR-2, and VEGFR-3 respectively and through these signaling pathways activate different cascades and exert their various biologic effects. 59

VEGF is now described as a multifunctional peptide, capable of inducing receptor-mediated endothelial cell proliferation and angiogenesis both in vivo and in vitro. 54, 56-58 The VEGF family is made up of at least five members whose effects are mediated via three VEGF receptors (VEGFR), (Figure 3).

These receptors communicate with the cell's interior via transmembrane receptor tyrosine kinases (RTKs). The VEGF gene is under intricate transcriptional regulation, 60 and due to alternative splicing of its pre-mRNAs, four different RNA isoforms are expressed with diverse biological properties. This process gives rise to the other family members of this class of cytokine- VEGF-B, VEGF-C, VEGF-D, VEGF -E, and platelet-derived growth factor (PDGF). 61-64

Pro-angiogenic factors, for example, VEGF, excites angiogenesis through the VEGF receptors (VEGFRs) and ligands (Figure 3). 65,66 The induction and progression of angiogenesis proceed in distinct steps during tumor development and can be observed through the action of vascular endothelial growth factor (VEGF) and acidic and basic fibroblast growth factors (FGF1/2). After it is expressed, VEGF binds to its receptor (VEGFR) and ligands located on the surface of ECs (Figure 3). 13,65,67,68 After binding to, and consequent activating the transmembrane tyrosine kinase receptors on the surface of the cell, it leads to dimerization, autophosphorylation, and activation of the downstream signaling pathway. This process is followed by the survival, proliferation, migration of the ECs, inhibition of apoptosis, and after several cascading processes, tube formation and sprouting. This process eventually, but slowly transforms into a developed network of new blood vessels. VEGF also induces vasodilation and stimulates vascular permeability, an underlying characteristic of tissue inflammation and the tumor microenvironment. 69

Increase in the production of pro-angiogenic factors, such as VEGF and proteolytic enzymes, 71 and the decrease in anti-angiogenic factors, 5,71,72 results in the activity of the ECs discussed above. Ultimately, a capillary network is successfully laid down that supplies the growing tumor with sufficient nutrients and oxygen. 73 Through this new vascular bed, the tumor cell, taking advantage of this trail, may enter the systemic circulation, and promote distant metastases. Therefore, the number of metastasis sites is positively correlated with the number of cancer cells entering the circulation initially. 74

Since its discovery, the number of angiogenic inducers identified has increased over the last decade and implicated in the regulatory process of angiogenesis in tumors. 75-77 Conceivably, tumor angiogenesis presents a uniquely attractive target therapy in all types of human tumors, and by interfering with the intracellular signaling of VEGF and VEGFR, anti-angiogenic therapy inhibits the growth of tumor vessels. 50,78-80

## Incidence of Angiogenesis in Breast Cancer

An angiogenic disease is described as either an excessive or deficient growth of microvessels. Initially, angiogenesis was implicated in cancer, arthritis, and psoriasis. However, the effect it has in several other diseases has been described. 81 The nature and composition of tumors make them inherently prime for effective angiogenic growth. An active vascular system is made up of adipose tissue, enveloped by stromal cells which gives it a supporting framework for the tumor's vascular system to emerge. White adipose tissue sustains the growth of the new vasculature and supports the development and progression of breast cancer in mouse models, 82 and the brown adipose tissue (made up of cells with numerous mitochondria) supports the tumor growth by providing a constant supply of oxygen and nutrients. 83 Both types of adipose tissues are responsible for producing angiogenic factors, most commonly VEGF A, B, and C, basic fibroblast growth factor (bFGF)/FGF-2; matrix metalloproteinases (MMPs); and IL-8, factors associated with breast cancer. 83,84 This aberrant growth of blood vessel has now been implicated in many life-threatening and disabling diseases conditions, such as cardiovascular disease, cancer, blindness, and diabetic ulcers. 9,49, 85-89

As already discussed, abnormal angiogenesis is also critical for cancer metastasis including breast cancer metastasis. 12, 90-93 Angiogenesis involves a coordinate regulation of some vascular growth factors, such as basic fibroblast growth factor (bFGF), transforming growth factor beta-1 (TGFβ-1), platelet-derived EC growth factor, placenta growth factor, and some other growth factors, 94-96 and clinical studies have shown that it plays a critical role in breast cancer progression and metastasis. 97 These growth factors are expressed and/or upregulated in aggressive human breast cancers, and among these growth factors, the expression of VEGF and its different isoforms has been characterized as the most significant in breast cancer, 98 although low-level protein expression has been detected in a healthy human mammary gland. 99

VEGF and IL-8 are the most studied growth factors. Breast cancer cell lines with high VEGF expression have been reported to also express high levels of interleukin-8 (IL-8), suggesting that they play very crucial roles in the promotion of angiogenesis in breast cancer angiogenesis, 100-103 A high level of VEGF receptor-3 has been detected in invasive breast cancer and also found to be upregulated in the endothelium of angiogenic blood vessels. 104 The interaction between VEGF-A and VEGFR-1 or 2 is intricately involved in breast cancer development, progression, and metastasis. 50,105-108

One of the prognostic indicators for survival is the level of angiogenesis in breast cancer. 21,22

An increased level of angiogenic growth factors in the breast cancer cells correlates with the aggressiveness and risk of the invasive breast cancer, 13, 23,24 and this has also been linked to p53 genes inactivation. 26 Furthermore, the number of microvessels in an invasive breast carcinoma from surgical samples may be a predictor of metastasis or relapse. 22

Studies reveal that, for tumor development and metastasis to occur, angiogenesis in the tumors must involve an interplay of some or all these growth factors- VEGF, interleukin 8 (IL-8), basic fibroblast growth factor (bFGF/FGF-2), and matrix metalloproteinases (MMPs). 35,109,110

Interleukins are a group of proteins and signal molecules, generally called cytokines, and first discovered in leukocytes. 111 They are secreted by cells as an immune response to various pathological stimuli. IL-8 is a member of the IL family that is produced by macrophages, airway smooth muscle cells, tumor cells, and other cell types. 112-115 and has been reported to excite the production of VEGF in ECs by binding with its receptor, and thereby activating VEGF receptors. 116 IL-8 also has a direct influence on angiogenesis by enhancing the proliferation and survival of EC, upregulating matrix metalloproteinases in certain EC lines, 110 and stimulating the formation of capillary tubes in vitro. All these are critical features of breast cancer progression and metastasis. 100-102,117-119 Furthermore, breast tumors with upregulated IL-8 levels have been observed to be more aggressive and invasive, making IL-8 levels an attractive target for anti-angiogenic treatments, 35,103 and a potential prognostic biomarker for various cancers, including breast cancer. 119

Fibroblast growth factors (bFGF/FGF-2) are collectively a family of powerful angiogenic stimulators linked to breast cancer risk. 120-123 Substances can modulate the interactions between FGF-2 and its receptor in the extracellular environment leading to regulation of angiogenesis, and subsequent tumor progression, and metastasis. 121,124-126 MMPs belong to a larger family of proteases 126 involved in angiogenesis due to their ability to degrade extracellular matrix proteins, and thereby remodel the extracellular matrix. They are primarily involved in destabilization of the existing blood vessel wall, degradation of matrix proteins, and migration of ECs-stages that have been described as the initiation process of angiogenesis. 127-129

Several antiangiogenic treatments that have been approved for clinical use target these pro-angiogenic growth factors, and/or their receptors, cytokines, and proteases associated with them. 130-134 Some of the examples of compounds/drugs that have gone through clinical research and approval will be covered in the next section.

## Inhibitors of Angiogenesis (in Breast Cancer) and their Modes of Action

Inhibiting the proliferation of the vasculature was proposed several years ago by Judah Folkman as a model for cancer treatment, 135 and this, researchers later discovered, would entail treatment with anti-angiogenic factors, or/and compounds that can decrease the release of pro-angiogenic factors, prevent their binding to receptors, or inhibit their actions. As a result, research has made the inhibition of the VEGF pathway a central focus of angiogenesis therapy. Some of the strategies that have been formulated, other than inhibiting the VEGF pathway, include employing antibodies targeting VEGF or VEGFR, use of soluble VEGFR/VEGFR hybrids, and use of inhibitors directed against tyrosine kinase. 136,137

### Endogenous Inhibitors of Angiogenesis

As discussed above, endogenous stimulators and inhibitors regulate angiogenic processes in the body. These are found in cells or systemic circulation as protein, glycoproteins, proteoglycans, or small proteins, where they interfere with specific activities in and by the EC, such as down-regulation of genes expression, cell formation and migration, and tube morphogenesis. 138

**Figure 4:**
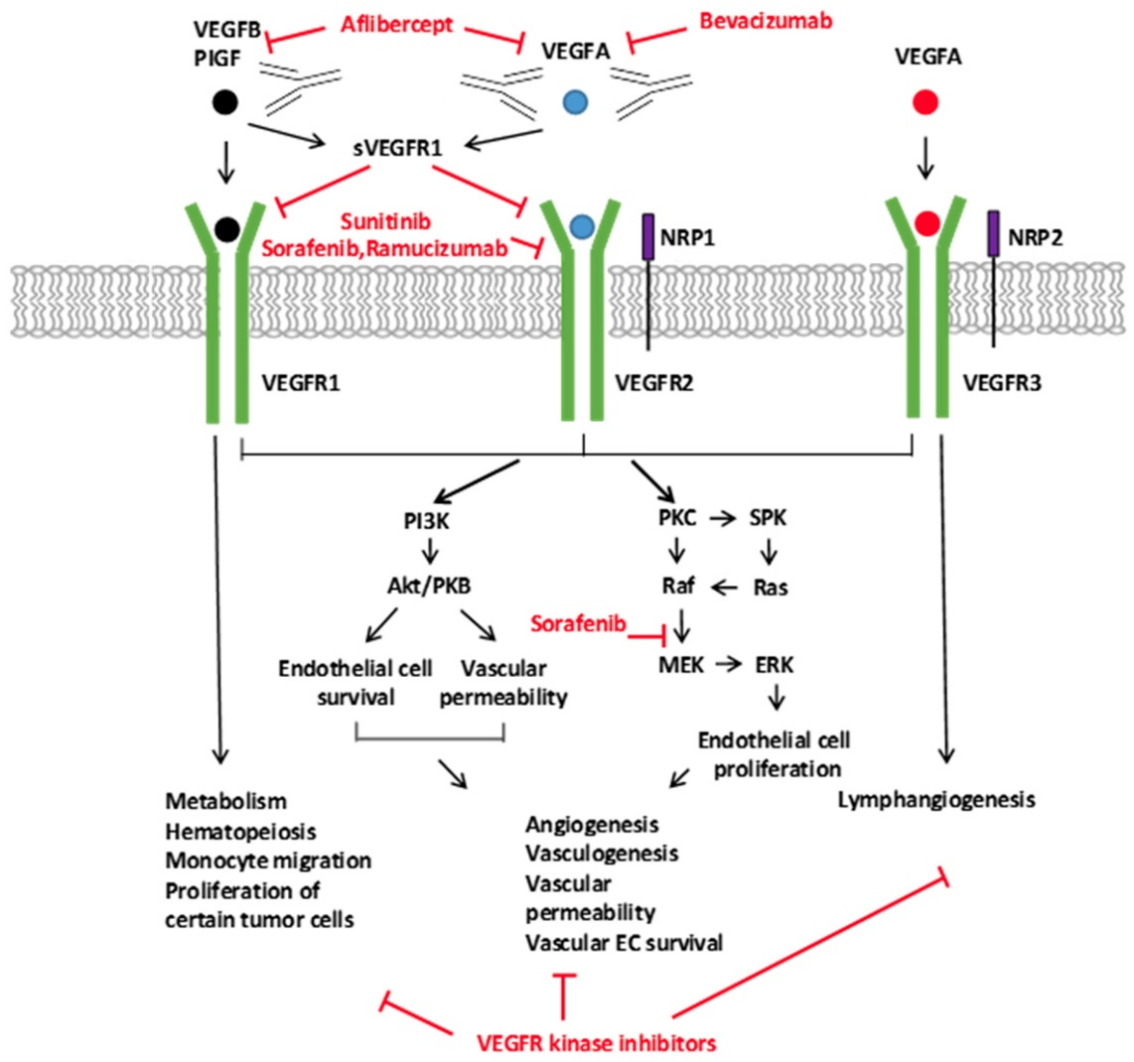
Signaling of the VEGF Ligands, Receptors, and the Inhibitors of the VEGFA Pathway. The VEGF family of ligands and their receptor-binding patterns are shown at the top. VEGF ligand family members, VEGFA, -B, -C, -D, and placental growth factor (PIGF), selectively bind to three tyrosine kinase receptors, VEGFR1, VEGFR2, and VEGFR3. As co-receptors of VEGFR2 and VEGFR3, neuropilin 1 and 2 (NRP1 and NRP2) modulate their signaling pathways. The soluble VEGFR1 (sVEGFR1) inhibits the signaling of VEGFR1 and VEGFR2 by sequestering free ligands. The different VEGF pathway inhibitors and their targets are indicated with red. Downstream VEGFR signaling pathways are shown on the bottom. VEGFR activates many proteins through PKC or PI3K. The activation of downstream signal transduction molecules leads to numerous distinct biological processes as indicated in the diagram. 139

Some of the endogenous inhibitors and a brief description of their mechanism of actions are discussed below; a list of several others is shown in Figure 4.

**Thrombospondins** (TSP). The thrombospondins (TSP) are a family of calcium-binding glycoproteins, composed of five highly conserved structurally related ECM proteins. They are secreted from the α granules of stimulated platelets and play a critical role in regulating in coagulation, cell adhesion, angiogenesis, and inflammation. In vetebrtates, this family is divided into two subgroups-A and B, and thrombospondins in subgroup: A (-1 and -2) are homotrimers. 140,141

Interestingly, Thrombospondin-1 (TSP-1) was the first protein to be identified as a naturally occurring angiogenic inhibitor. 41 Studies have proposed that TSP-1, depending on proteases that produce the fragments of TSP-1, may display a dual nature, i.e., antiangiogenic and proangiogenic. 142,143 TSP-1 has been reported to inhibit tumor growth and metastasis, therefore qualifying it a powerful inhibitor of neovascularization and tumorigenesis in vivo. 144-146. Numerous research indicates that TSPs found in breast cancer, can function as strong endogenous antiangiogenic factors, consequently leading to tumor suppression; 144,147-151 TSP-2, which is similar structurally to TSP-1, has a comparable antiangiogenic and antitumor property. 152,153

Scientists have proposed, as possible mechanisms for this antiangiogenic activity, the inhibition of VEGF using thrombospondin-2, which consequently prevents EC migration, tube formation, and EC-specific apoptosis. 154

**Endostatin**. Endostatin, a fragment of collagen XVIII produced by tumor cell proteases, blocks EC proliferation and migration. 155-158 It is considered a potent antagonist of angiogenesis and inhibitor of tumor growth in mouse models. In experimental animal models, recombinant endostatin effectively inhibits angiogenesis and suppresses primary tumor growth and metastasis without obvious side effects or toxicity. 155,159,160 It is reported to have several possible mechanisms of action in relation to the inhibition of tumor angiogenesis including inhibition of tumor necrosis factor alpha (TNFα), initiation of the JNK signaling pathway, 160 interacting with and antagonizing alpha 5- and alpha(v)-integrins on human endothelial cells surface, 158 and inhibiting EC cycle progression. 157

Endostatin inhibits signal transduction that is stimulated by FGF-2, thus, among other actions, blocks EC motility, 162 and inhibits signaling mediated by VEGF through direct interaction with the subfamily of receptor tyrosine kinases (RTKs), VEGF-R2/KDR/Flk-1, in HUVECs. 163-165 It was found to also reduce the growth of certain breast cancer cells in vivo and thereby might prove effective in the treatment of breast cancer. 166

**Interferons.** (IFNs) are a group of cytokines that mount a cellular immune response to a range of pathogens including viruses, bacteria, and tumors. 167 Studies show that they inhibit angiogenesis, induced by tumor cells in mice, by significantly lowering the secretion of the major angiogenic factor produced by tumors: interleukin (IL)-8. 168,169 Previous research reveals that interferons-α/β can inhibit tumor angiogenesis by downregulating translational expression of bFGF/FGF-2 and reducing expression of the MMP-2 gene, making them potential candidates for antiangiogenic treatments. 166,170-175 Other research, however, suggests the mechanism of IFN-α is not mediated by bFGF or VEGF. 176-178

**Interleukins.** (ILs) are a family of leukocyte-derived cytokines, involved in cell signaling, predominantly serving as modulators of the immune responses. 179,180 They also have broad-ranging effects on various physiologic activities, including angiogenesis. Members of this family exert their antiangiogenic properties in diverse ways. IL-1β inhibits FGF-stimulated angiogenesis via an autocrine pathway, 181 IL-4 induced angiogenesis by inhibiting bFGF, 182 while IL-12 blocks angiogenesis through the downstream chemokines, such as IFN-inducible protein-10 and IFN-γ- induced monokine. 183-185

**Angiostatin.** Angiostatin is an endogenous angiogenesis inhibitor found in humans and several other animal species. It is a proteolytic fragment of plasminogen that was isolated from tumor-bearing mice. 186 Studies show that angiostatin suppresses tumor metastasis by inhibiting the formation of blood vessels and is believed to inhibit endothelial cell migration and block tumor progression, 157, 186-188 although the fundamental mechanisms remain uncertain. 187

Continous exposure of cells to angiostatin has been revealed to block the activation of the MAPK, extracellular-signal-regulated kinases-1 (ERK1) and ERK2, by FGF-2 or VEGF in human in endothelial and smooth muscle cells, 188,189 which ultimately leads to disruption of angiogenesis.

**Decorin**. Decorin is a member of a small leucine-rich proteoglycans family that is secreted by mesenchymal cells, connective tissue cells, and tumor stromal cells. It is involved in numerous cellular processes including wound healing, matrix organization, the formation of collagen fibrils, and maintenance of cell proliferation by interacting with growth factors and other ECM proteins. 190-195 It influences the balance of anti- and proangiogenic proteins, thereby creating a pathological environment. It is an inhibitor of tumor angiogenesis, progression, and metastasis through its association with certain receptors and proteins such as EGFRs, TGF-β, VEGF and VEGFR-2, and bFGF/FGF-2. 196

Decorin also inhibits tumor cell-mediated production of hypoxia-inducible factor-1α (HIF-1α), and c-Met, and concurrently stimulates the rapid production of the antiangiogenic, angiostatic molecules thrombospondin-1 and tissue inhibitor of metalloproteinases 3 (TIMP3). 196-198 It induces Peg3-dependent autophagy in both microvascular and macrovascular endothelial cells, which leads to angiogenesis suppression. 199 Also, decorin significantly inhibits VEGFR2 by binding directly to a site that incompletely overlaps with the site for VEGF-A. 200, 201

### Pharmaceutical Inhibitors of Angiogenesis

Molecules targeting angiogenesis pathway have been extensively researched in the treatment of breast cancer and other forms of cancer. 26 Few key clinical strategies in combating angiogenesis have been adopted, including- the use of monoclonal antibodies binding VEGF, the use of decoy receptors, the use tyrosine kinase inhibitors, and the use of monoclonal antibodies targeting VEGF receptors. 202-206 Bevacizumab and ramucirumab are monoclonal humanized antibodies designed to inhibit the interaction between VEGF ligands and receptors. 207,208

These agents are used in breast and other types of cancer therapy. 209 Several anti-angiogenesis drugs have been approved by the FDA and are currently used in cancer treatment. Still, other agents are in different stages of clinical development or preclinical evaluation. 210

In 1993, results showed that bevacizumab, a humanized monoclonal antibody, and called Avastin, binds specifically to all isoforms of VEGF-A and disrupts its activity and interactions with VEGFR-1 and -2, leading to a decrease in tumor growth. 211,212 (Figure 4). 213

Shortly, after that in 2004, it was approved by first US Food and Drug Administration (FDA) for the treatment of advanced colorectal cancer, then other forms of cancer, either alone or together with other chemotherapeutics, 214,215 and then extended for use in several others, including breast cancer. 216-218

In 2007, the European Medicines Agency (EMA) granted the use of bevacizumab in metastatic HER2-negative breast cancer, followed by the approval of the FDA in 2008 for the same indication. 219

**Figure 5:**
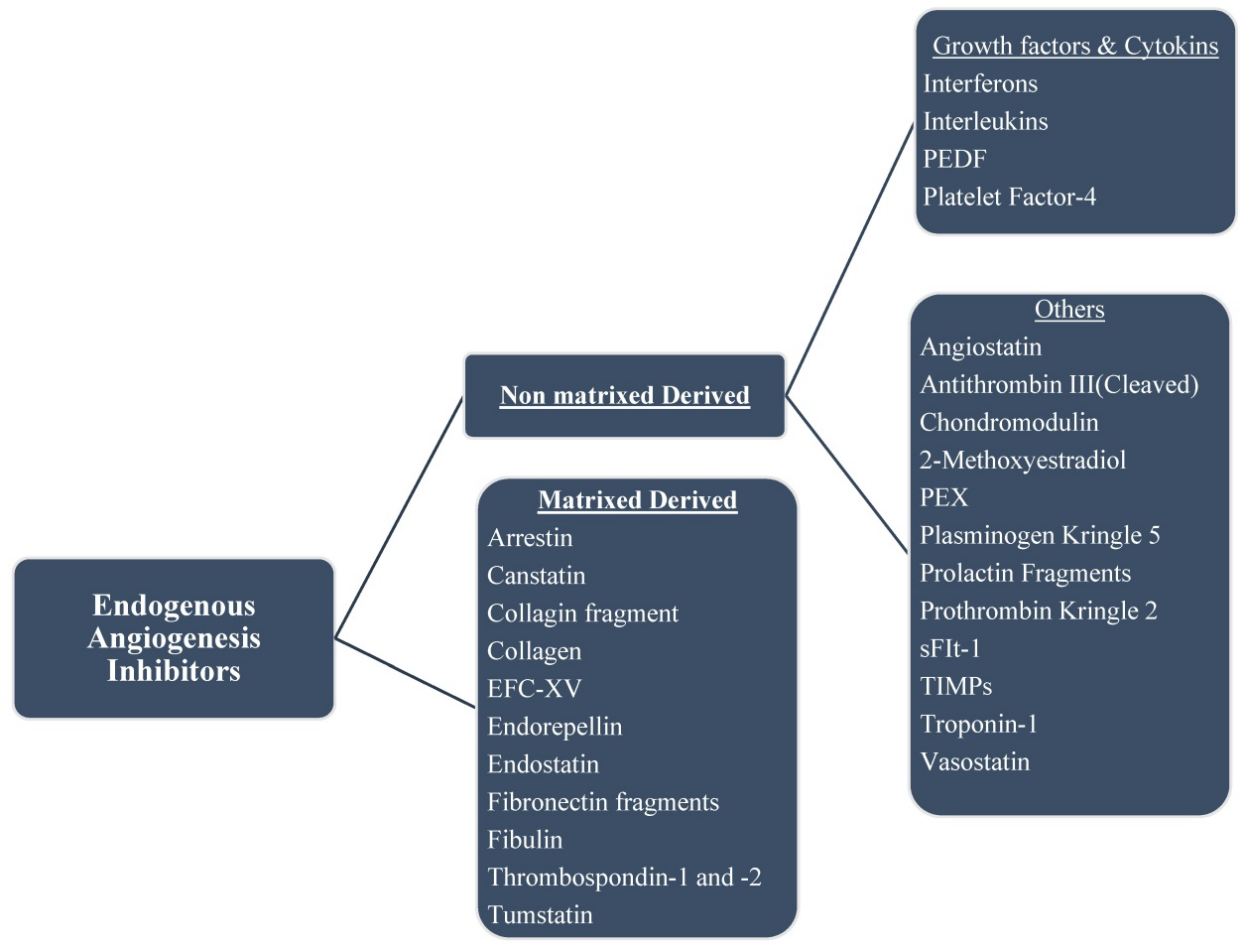
Chart of endogenous angiogenesis inhibitors. Several are fragments of a naturally occurring extracellular matrix (ECM) and basement membrane proteins. The endogenous inhibitors of angiogenesis are divided into two major categories.

The clinical breakthrough of bevacizumab inspired the development of several VEGFA pathway inhibitors, including a monoclonal antibody against VEGFR2 that inhibits the binding to a ligand, a recombinant chimeric receptor that sequesters various ligands in the VEGF family, and several small-molecule tyrosine kinases that inhibits the action of VEGF receptors (Figure 5). 220

Several of these compounds have been proven to have positive results with patients with various forms of cancers, 28, 29, 30 and have been approved by the FDA and/or EMA. 28-30 While we briefly discuss several of these anti-angiogenesis drugs, we particularly highlight the current findings that show the activities of antiangiogenic agents in the treatment of breast cancer.

The drugs and treatment strategies highlighted here do not represent standard therapies for only breast cancer patients, nor does it seek to exhaust all currently used drugs or regimen in the treatment.

The FDA lists the following as approved angiogenesis inhibitors:

Axitinib (Inlyta®)

Bevacizumab (Avastin®)

Cabozantinib (Cometriq®)

Everolimus (Afinitor®)

Lenalidomide (Revlimid®)

Lenvatinib mesylate (Lenvima®)

Pazopanib (Votrient®)

Ramucirumab (Cyramza®)

Regorafenib (Stivarga®)

Sorafenib (Nexavar®)

Sunitinib (Sutent®)

Temsirolimus

Thalidomide (Synovir, Thalomid®)

Vandetanib (Caprelsa®)

Ziv-aflibercept (Zaltrap®) 221

Anti-VEGFR antibodies

One of the earliest and extensively researched strategies in combatting antiangiogenic treatment is the use of a monoclonal antibody neutralizing circulating VEGF. They involve monoclonal antibodies such as bevacizumab, antibody derivatives such as ranibizumab (Lucentis), or orally-available small molecules that inhibit the tyrosine kinases stimulated by VEGF: lapatinib, sunitinib, sorafenib, axitinib, and pazopanib. Some target VEGF receptors rather than the VEGFs and have been especially attractive in the triple-negative breast cancer subtype because they were shown to increase VEGF expression and enhance angiogenesis. 26

**Bevacizumab (Avastin).** Bevacizumab is a humanized, monoclonal antibody that binds VEGF-A and blocks its activity and interactions with VEGFR-1 and -2, reducing tumor growth. 163, 211,222-224 Incidentally, it was the first FDA approved angiogenesis inhibitor and the first commercially available anti-angiogenesis drug, used in combination with additional chemotherapeutics for treatment of metastatic colon cancer. 215,225 Later, this was expanded with its single and combinatorial use for non-small cell lung cancer, renal cell carcinoma, pancreatic cancer, ovarian cancer, advanced kidney cancer, glioma, leukemia, and breast cancer. 215,218, 226-228

In phase, I study, bevacizumab was tested in combination with chemotherapy and reported a good safety profile, 228 then got expanded as the first line of treatment when combined with paclitaxel, a chemotherapy medication, in breast cancer patients due to its ability to double the median progression-free survival (PFS). 216 In combination with other chemotherapy therapies, it was shown to have significant positive outcomes in patients with different respective tumor types. 217,229,230

However, the therapeutic value of bevacizumab in this indication was called into question when it failed to show an effect on overall survival. Furthermore, it produced an array of side effects, including high blood pressure, significant excreted protein levels, bleeding, and blood clots. 132, 231, which seemed to increase when combined with chemotherapy. 215,232,233

Eventually, in 2011, the FDA revoked the approval for the therapeutic use of bevacizumab for metastatic breast cancer due to lack of substantial improvements in response and survival. 27

**Cetuximab (Erbitux®).** Cetuximab is a monoclonal antibody that binds to the extracellular domain of epidermal growth factor receptor (EGFR), blocking the subsequent binding of the ligand, and stimulation of the receptor. This leads to the degradation of the receptor and consequent inhibition of cell proliferation and angiogenesis. It is the first monoclonal antibody that was approved by the FDA as a second-line treatment for metastatic colorectal cancer 234, 235 and was shown to downregulate the VEGF in human colorectal carcinoma (CRC) cell line and in human CRC mouse xenografts. 236

**Ramucirumab.**Ramucirumab is a human monoclonal antibody that binds to VEGFR2, blocking the binding of the receptor and its ligand VEGF. Presently, ramucirumab has EMA and FDA approval for treating advanced gastric cancer, metastatic colorectal cancer, and advanced non-small cell lung cancer. 237-239 It presents itself as a receptor antagonist blocking the binding of vascular endothelial growth factor (VEGF) to VEGFR2 by binding to VEGFR2. Preclinical and clinical evidence suggests that Ramucirumab may critical roles in angiogenesis in breast cancer growth, invasion, and metastasis. 240

**Ranibizumab.** Ranibizumab is a recombinant, humanized, monoclonal antibody Fab fragment against VEGF-A. The antibody fragment was generated from the same parent mouse antibody as bevacizumab and approved as an anti-angiogenic drug for the treatment of the "wet" type of age-related macular degeneration. 241, 242

### Tyrosine kinase inhibitors

Another strategy employed in targeting the VEGF signaling is inhibiting the signal transduction, particularly of the VEGF-receptor, with tyrosine kinase inhibitors (TKI). 204,205

Tyrosine kinases play a vital role in modifying the signaling of growth factor. When forms of these enzymes are activated, it can cause increases in proliferation and growth of tumor cell, induction of antiapoptotic effects, and promotion of angiogenesis and metastasis. 243

TKIs are small-molecular-weight drugs that block the kinase activity of various receptors. TKIs cross the cellular membrane, where they can interfere with downstream signaling pathways. 244 The FDA has approved almost 30 small-molecule kinase inhibitors, including agents that target VEGFR, e.g., sunitinib, sorafenib, axitinib, and pazopanib.

Unlike VEGF neutralizing antibodies, TKI does not interfere with the binding of VEGF to its receptors, and they usually target other kinases, besides, such as PDGFR, FGFR, and c-KIT. 245 Currently, they collectively serve more as a secondary or tertiary line of therapies, rather than as primary therapy, and probably more useful in combination with traditional cytotoxic chemotherapy. 246 Some of these small molecules have been discussed below.

**Axitinib (Inlyta).** Axitinib is the first receptor tyrosine kinase inhibitors (TKI) compound with established antitumor activity. In clinical trials, it was shown to incite some response in treating renal cell carcinoma, 247 and several other types of tumor, 248 either alone or used in combination with chemotherapeutic drugs. 249-254

Axitinib is a small molecule inhibits VEGF and platelet-derived growth factor receptor (PDGF), thereby inciting an anti-angiogenic effect. It was reported to be a potent and selective inhibitor of VEGFR-1, -2, and -3, and has been reported to reduce vascular permeability, tumor vascularization, and tumor volume. 251 In animal (xenograft) models, it was shown to significantly inhibit the growth of breast cancer. 255

Clinical studies, however, indicates that Axitinib has shown significant benefits only in patients who have previously received paclitaxel. 256

**Sorafenib (Nexavar®**). Sorafenib is a TKI with a different mechanism of action that acts downstream targeting VEGFR-1, -2, and -3, PDGFR-β, and Raf-. 257,258 It received FDA approval for the treatment of inoperable hepatocellular carcinoma, 259 advanced renal cell carcinoma, 260, 261 and for recurrent or metastatic thyroid carcinomas. 262,263

However, the results from the treatment were mixed, since the tumors progressed in some patients. 263 Based on the NCI clinical trials search results, there have been at least 168 active clinical trials involving sorafenib in different cancers, and some are still ongoing, making it one of the recurrently studied drugs. 264-266

The clinical result of sorafenib in breast cancer regimen is encouraging, although limited, with completed phase I/II trial of the combination of sorafenib and anastrozole, suggesting that sorafenib may be able to delay the initiation of chemotherapy in some patients, although this combination nevertheless, had significant toxicity. 267

However, the combination of sorafenib with capecitabine, either as a primary or secondary line of treatment, did not improve PFS, OS, or ORR, and furthermore, the rates of toxicities were significantly higher with sorafenib alone. 268

**Sunitinib (Sutent®)**. Sunitinib is another TKI that was approved by the FDA and EMA to treat renal cell carcinoma (RCC), and imatinib-resistant gastrointestinal stromal tumors, and well-differentiated pancreatic neuroendocrine tumors (pNET). 269 When activated, it inhibits VEGFR and PDGFR, 270 and has been evaluated in about 150 clinical trials for several cancer conditions, such as ovarian, 270 breast, 271 and non-small cell lung cancer, 272 among others, 273 with mixed results. 274-277

Currently, the benefit of sunitinib in breast cancer is still not very clear, due to some disappointing results observed. It displayed a single-agent reaction in the treatment of metastatic breast cancer, 278 while in other trials, it failed to show a positive therapeutic result in either first-line or refractory breast cancer. 279-281

Also, three phase III studies investigating the effect of using a combination of sunitinib with chemotherapy, 280-282 and one comparing sunitinib versus capecitabine as monotherapy for patients, did not show improvement in PFS or OS. 284

**Cabozantinib (Cometriq®).** Cabozantinib is a TKI, a small molecule that targets and binds to the tyrosine kinase receptors, inhibiting the activity of multiple tyrosine kinases, including RET, MET, and VEGF, and consequently inhibiting tumorigenesis, angiogenesis, metastasis, and drug resistance. 285,286

Some studies suggest that cabozantinib is a potential agent for inhibiting tumor angiogenesis and metastasis in cancers, and the observed antitumor activity is the result of mechanisms affecting tumor angiogenesis and the inhibition of invasive tumor growth rather than the result of directly targeting cellular proliferation, 287 and clinical studies with patients with metastatic breast carcinoma patients revealed very promising data. 285

### Inhibitors of mTOR

mTOR, the mammalian target of rapamycin or the mechanistic target of rapamycin, plays a role in tumor cell proliferation and angiogenesis. 286 Rapamycin and related mTOR inhibitors block the expression of VEGF on endothelial cell, together with VEGF-induced endothelial cell proliferation. 288 mTOR inhibitors are a vital group of anti-angiogenic compounds, some of which include deforolimus, everolimus, rapamycin (sirolimus), and temsirolimus. 289,290

Research and preclinical work conducted earlier have shown that stimulation of the mTOR signaling pathway is linked with resistance to hormonal therapy in ER+ breast cancer, and drugs that inhibit mTOR signaling can contribute in overcoming this resistance. 291-293

**Temsirolimus (Toricel®).** Temsirolimus (Toricel®) is a mTOR inhibitor, approved for treating advanced renal cell carcinoma, 294 and treatment with this compound inhibits tumor angiogenesis, among other activities, by downregulating the synthesis of VEGF. 295

## Other Angiogenic Agents

**Bortezomib (Velcade®).** Bortezomib is a proteasome inhibitor that causes cell death and tumor regression by interfering with cancer cells signaling. 296-298 It has indirect anti-angiogenic properties, 299 and FDA-approved for the treatment of myeloma that has relapsed following two previous therapies. In a clinical trial, patients with metastatic breast cancer were treated with bortezomib, and although it was well tolerated, it showed limited clinical activity against metastatic breast cancer when used as a single agent. 300

### The VEGF-Trap

**Ziv-aflibercept (Zaltrap®).** Aflibercept is a drug, approved by the FDA and EMA for the treatment of wet macular degeneration for metastatic colorectal cancer as an inhibitor of VEGF. 301-303, It binds to VEGF in circulation, functioning like a soluble VEGF decoy receptor, inhibits the activity of the VEGF-A and VEGF-B, as well as PGF, 304 and consequently inhibiting the growth of new blood vessels in the choriocapillaris or the tumor, respectively. 305-307

Wu, et al, in their research suggest aflibercept as another potential VEGF pathway-targeted antiangiogenic agent in combination with neoadjuvant-plus-adjuvant chemotherapy regimens in triple-negative breast cancer (TNBC). 308

### Natural Inhibitor of Angiogenesis

**Neovastat.** Neovastat is an extract from shark cartilage that inhibits angiogenesis by selectively competing for the binding of VEGF and its receptor, inhibits matrix metalloproteinases, incites tissue plasminogen activator enzymatic activities, and induces apoptotic activity in ECs. 309 Neovastat was found to have significant antitumor activity, 310,311 and in vivo experiments revealed that it inhibits the formation of blood vessels in Matrigel implants containing basic fibroblast growth factor (bFGF). 312

## Anti-Angiogenesis Therapies in Bca: Past, Current, Future Explorations

The reasoning underlying antiangiogenic research and motivation as an active area of investigation was the idea that inhibiting the formation of a blood vessel in tumors would induce tumor dormancy or regression by depriving them of necessary oxygen and nutrients. Although clinical results provided sufficient evidence that anti-angiogenic treatment is a valid therapeutic strategy, the full potential of this approach seems to have been inadequate. The use of these agents (discussed above and several others) in combination with standard protocols or neoadjuvant therapy targeting angiogenesis -driven tumor growth have shown average clinical results.

One of the earliest preclinical research revealed that treatment with an anti-VEGF monoclonal antibody yielded a significant decrease in vascular density and tumor growth delay in mice. 313 However, these clinical trials did not translate into the same expected results in solid human tumors-reporting just moderate response rates and little survival gains for patients. 313

Following several years of clinical research, anti-VEGF agents seem to be effective as single agents only in a few types of cancers, while effective in others, such as breast cancer, when combined with chemotherapy. 313,314

So far, studies have reported that a combination of antiangiogenetic compounds with standard chemotherapy treatments in metastatic breast cancer has produced a limited clinical impact on overall survival in several forms of cancer, including breast cancer.

Few years after its approval, several controversies arose regarding the use of bevacizumab in the treatment of metastatic breast cancer. Although the more than 2000 trials revealed that combining bevacizumab with paclitaxel improved PFS, as well as overall survival rates compared to paclitaxel alone, it revealed a range of side effects including bleeding and blood clots, abnormal excreted protein levels, and high blood pressure. 31,314 However, after the FDA eventually withdrew the approval of bevacizumab for metastatic HER2 negative breast cancer, 32,315 the EMA did not follow suit immediately because of the increased PFS the drug in combination with capecitabine or paclitaxel showed. 316

Enormous challenges persist in this area of research, in the ensuing clinical trials, and eventual treatment. The challenges that surround developing antiangiogenic agents and determining their efficacy, however, are not unique to breast cancer treatment. If the goal is to optimize the possibility of these agents ultimately making an impact in the treatment of breast cancer, then researchers must anticipate and address the potential obstacles. Several factors may explain the low efficacies observed so far, including toxicity, drug resistance, alternative mechanisms of angiogenesis, and the challenge of identifying the right patient who will benefit from anti-VEGF therapy in breast cancer. Some of these challenges and possible strategies to overcome are discussed next.

**Toxicity.** Since angiogenesis is not a very common process in adults, VEGF-targeted treatments were expected to be toxicity free. Several clinical trials, however, revealed that anti-angiogenic therapy had numerous adverse side effects. 202 While some of these can be appropriately managed with proper care, increasing levels of toxicity could lead to an intermittent treatment regimen, reduction in dosage, or treatment cessation, ultimately or possibly minimizing the efficiency of the treatment followed by rapid regrowth of the tumor. However, several studies have proposed using some of these side effects as a predictive biomarker for treatment efficiency, due to observation such as one reported between hypertension and longer PFS and/or OS in patients undergoing anti-angiogenic treatment. 317-320

**Resistance.** Resistance to antiangiogenic treatment is a critical issue. Since normal endothelial cells are genetically stable, it was presumed initially that antiangiogenic treatment would not face the problem of resistance. 321 However, some forms of cancer have been reported to manifest both evasive and intrinsic resistance to anti-angiogenic therapies. (206,v322) This problem is one of the contributing factors to the low efficiency of the anti-VEGF therapy in breast cancer and understanding the mechanisms behind this process could enhance the care and contribute to treatment.

**Heterogeneity.** Within the endothelium vessels involved in angiogenesis, significant heterogeneity and frequent mutations have been described. 323 This has presented several challenges for effective and proper treatment; for instance, different mechanisms may be involved in the vascularization within the same tumor at a specific time and between different tumors in the same patient, 324 which has been reported as responsible for the variation in drug response/ drug metabolism or transport. 323

This heterogeneity has been described in invasive cancers, which also commonly express multiple angiogenic factors. Several different pro-angiogenic proteins have been identified in primary breast tumors. 99 and the heterogeneity may also be a major contributing factor to drug resistance and low efficacy of the anti-angiogenic therapy in breast cancer and all other cancer therapeutics. 325,326

**Biomarkers.** A biomarker can be described as "a characteristic objectively measured and evaluated as an indicator of normal biologic processes, pathogenic processes, or pharmacologic responses to a therapeutic intervention." 327,328 Due to the complexity in the mechanisms in determining response and resistance to anti-angiogenic drugs, it has been difficult to come up with predictive biomarkers for this class of agent. Several pro-angiogenic proteins mentioned above, including VEGF, has been speculated to be used indirectly as biomarkers to measure angiogenic activity in breast cancers. One of the early biomarkers to be examined was the plasma concentration of VEGF-A, 329 and higher levels of some of these proteins have been found in the serum and urine of cancer patients. 330

Therefore, plasma levels of VEGF-A or VEGFR-2 continue to serve as promising biomarkers. 331-333 Some of the ways biomarkers can be used include for prognosis (for assessing the overall disease outcome), 334 predictive (to provide data about the response or survival of a certain patient under a specific treatment before therapy), 335 screening, and diagnostic.

Furthermore, the increase in the discovery of more biomarkers uncovers an equally important challenge of standardizing procedures for assessing the biomarkers across different centers for validation and routine adoption. 202 Consequently, the probability appears low that a single biomarker would suffice to effectively predict the efficiency of anti-angiogenic agents, particularly in cases with multiple metastases, where challenges posed by the tumor heterogeneity will complicate the attempt. 202 Extensive studies conducted is yet to produce a biomarker for the efficacy of bevacizumab in different patients, which may explain in part its inability to produce significant benefits clinically. 336,337

Ongoing research into factors such as levels of pretreatment serum VEGF is attracting some attention as having a prognostic role. 31, 338, 339 Patients that had higher than normal levels at baseline were reported to have a poorer prognosis and an increased treatment effect of bevacizumab when compared with patients with low levels. 31,338 The proposal that VEGF level, in serum or tumor biopsies, could fulfill the requirements of a predictive biomarker has so far remained inconclusive. 340,341

Although circulating VEGF levels in cancer patients revealed the importance of VEGF, not as a predictive biomarker, but a prognostic biomarker. 202 Additionally, as mentioned above, some of the adverse side effects related to antiangiogenic drugs seems to be positively linked with response to treatment. For instance, hypertension associated with the bevacizumab or TKIs has been reported to be correlated with clinical response in patients with breast and some other form of cancer. 342

The use of biomarkers offers some hope as a possible solution to the problem of indiscriminate anti-angiogenic therapy, by providing physicians a better way of selecting patients with the most significant possibility for a positive response to a treatment, which will also serve to address the problem of the overall high variability in the response of patient to anti-angiogenic drugs and the quick development of resistance by predicting patients' response to VEGF-inhibition and help individualize drug and determine dosage. 78 The search for such biomarkers is still ongoing, and numerous prospective candidates have been considered.

### Alternative approaches

Alternative strategies have been proposed in targeting vascularity of tumors. One such idea is to improve the delivery of chemotherapy by designing treatment that normalizes the tumor vasculature explicitly. 343-345 Preclinical and clinical studies have revealed that there is quick regrowth of the tumor due to rapid revascularization when antiangiogenic therapy is stopped abruptly. 346-348

An argument has been put forward that to develop a successful anti-angiogenic therapy in the future; a better appreciation will be needed of how different tumors attain vascularization and avoid the action of anti-angiogenic therapy. This could lead to the development of new anti-angiogenic strategies directed towards specific cancer. 202

However, the precise approach of how this would be effectively done in different cancers to suppress the tumor, prevent resistance, and prolong the survival of the patient is still being determined. 202

## Conclusion and Future Perspective

The increased angiogenesis, a measure of levels of VEGF or microvessel density, is an independent adverse prognostic factor in early breast cancer. 33 Tumors that are starved of blood supply stop growing and become necrotic, but if the connection to systemic circulation is established and sustained, it leads to hematogenous metastatic spread cancer. 8,33 Therefore, there is a practical justification for the use of anti-VEGF (specifically) and antiangiogenic therapies (in general) in primary, locally advanced and metastatic breast cancer.

Anti-VEGF treatment in metastatic breast cancer came with high hopes and excitement initially. One of the prominent results of the research was bevacizumab, which was received FDA approval based on very promising clinical trials. However, conflicting subsequent results and reports on adverse side effect led to the FDA and EMA revoking this approval. 349

The problem of drug resistance in angiogenesis treatments of many cancer types has been a persistent challenge, and several proposals have been offered including dugs or strategies targeting microenvironment tumor heterogeneity and microvascular heterogeneity. 350 Targeting the multifaceted nature of the disease, would require as many paths as can be unraveled in the developmental stage. However, several agents that multi target this disease has not revealed much effectiveness. 351 Some scientists have suggested that nanotechnology provide the best prospects of discovering agents that will enhance the treatment of these cancer types, and Nanoparticles (particles less than 100 nm) is expected to achieve the positive results that anti-angiogenic agents have struggled to attain, and to serve as strategy to reduce the challenge of drug resistance 352-353

Although most of the antiangiogenic drugs have not been very effective against breast cancer, the research leading to their discovery and subsequent clinical trials have provided a good foundation and direction for identifying prospective drugs, drug targets, and a better understanding certain mechanisms involved in the impairment of tumor angiogenesis, growth, and migration.

So, currently, the best strategy may be to take advantage of what we understand from the activities of the angiogenic agents in the early clinical studies and redirect our efforts from designing comprehensive treatments for a population, to targeted therapies for specific patients or groups of patients. The excitement observed some years ago may appear to be on a downward slope, but some exciting discoveries, however, still get often published that keeps a cautious optimism alive.

Recently, immune checkpoint inhibitors (ICIs) have been increasingly studied alongside angiogenesis. Combination treatment with these two approaches have promising results in a wide variety of cancer types, including hepatocellular carcinoma, NSCLC, and melanoma [354-356]. ICIs are molecules that are able to activate anti-tumor responses by blocking negative regulatory immune signals [357]. Some common molecules include programmed cell death protein 1 (PD-1) and cytotoxic T-lymphocyte antigen-4 (CTLA-4) [354]. Pembrolizumab is an inhibitory antibody that targets PD-1, and the FDA has approved its use in 2017 as the first drug to be categorized not by cancer type, otherwise known as 'histology agnostic' [358]. Another drug that targets immune checkpoint inhibitors and their signaling pathway is nivolumab, which has also been approved by the FDA [356].

Immune checkpoint molecules serve an integral role in downregulating the magnitude of immune responses [357]. Upregulation in immune checkpoint signaling pathways like the PD-1/PD-L1 pathway can shield cancer cells for immune detection. Thus, immune checkpoint molecules are ideal targets for anti-cancer treatment. The PD-1 pathway is normally a T-cell inhibitory pathway, which is induced by binding of the PD-1 receptor on the cytotoxic T-cell plasma membrane to PD-L1 on the tumor [356]. The anti-PD-1/PD-L1 therapy enhances cytotoxic ability by restoring T cells from exhausted status [357].

There have been numerous studies done on the synergistic effect of anti-angiogenesis and ICI combination treatment in colon adenocarcinoma, kidney tumors, and mammary tumors in mice [357]. Several mechanisms have been found that relate to synergistic effect. VEGF can prevent lymphocytes from mobilizing via its effect on the Fas ligand [356]. Using a combination treatment of anti-VEGF and anti-PD-L1 in mice, a high proportion of exhausted T cells can be reversed [357]. In another study, bevacizumab (anti-VEGF) and atezolizumab (anti-PD-L1) were used in a renal cell carcinoma model [354]. This combination treatment increased the expression of MHCI in tumor cells and effector T cells, resulting in increased antitumor effects [354].

A strategy must be developed to select those patients that would benefit more from one particular antiangiogenic agent and determine when would be the most effective window of opportunity they are likely going benefit from the treatment. In this regard, although several scientists believe that more efficient drugs or treatment regimens designed to combat breast cancer angiogenesis may be further in the future, it is necessary to pursue the discoveries of potential new drugs and to determine the possibility finding new functions for previous ones.
